# Higher baseline TSH levels predict early hypothyroidism during cancer immunotherapy

**DOI:** 10.1007/s40618-021-01508-5

**Published:** 2021-02-12

**Authors:** C. Luongo, R. Morra, C. Gambale, T. Porcelli, F. Sessa, E. Matano, V. Damiano, M. Klain, M. Schlumberger, D. Salvatore

**Affiliations:** 1 Department of Public Health, University of Naples “Federico II”, Via S Pansini, 5, 80131 Naples, Italy; 2 Department of Clinical Medicine and Surgery, University of Naples “Federico II”, Naples, Italy; 3 Department of Advanced Biomedical Sciences, University of Naples “Federico II”, Naples, Italy; 4 Department of Endocrine Oncology, Gustave Roussy, University Paris-Saclay, 94805 Villejuif, France

**Keywords:** Immunotherapy, Hypothyroidism, Anti-thyroid antibodies, Thyroid immuno-related adverse events

## Abstract

**Background and purpose:**

Immune checkpoint inhibitors (ICIs) are monoclonal antibodies that enhance the immune response against cancer cells. ICIs are generally well tolerated, although endocrine immune-related adverse events (irAEs) are common. We investigated the risk factors for thyroid irAEs in patients treated with ICIs. Moreover, we evaluated the clinical outcome of subjects who became hypothyroid compared to euthyroid patients.

**Patients and methods:**

We retrospectively analyzed a series of 195 consecutively subjects treated with ICIs for metastatic tumors at the University of Naples “Federico II” between January 2014 and March 2020. Only subjects tested for thyroid function before and during the treatment with ICIs were included.

**Results:**

In the 96 patients treated with ICIs who were included [66 males, median age: 62 years (27–87)], thyroid irAEs occurred in 36 (37.5%), 16 (16.7%) a transient thyrotoxicosis, and 20 (20.8%) an hypothyroidism (in nine subjects hypothyroidism was preceded by a transient thyrotoxicosis). Only baseline TSH levels above 1.67 mIU/L and positive anti-thyroid antibodies (Ab-T) were associated with a higher risk of hypothyroidism. Patients with hypothyroidism during ICI treatment showed an improved 2-year PFS (HR = 0.82 CI 0.47–1.43; *p* = 0.0132) and OS (HR = 0.38 CI 95% 0.17–0.80; *p* = 0.011) compared to euthyroid patients.

**Conclusions:**

Baseline TSH levels above 1.67 mIU/L and presence of Ab-T are risk factors for the development of thyroid irAEs. Patients affected by thyroid irAEs showed a longer survival than patients who remained euthyroid.

**Supplementary Information:**

The online version contains supplementary material available at 10.1007/s40618-021-01508-5.

## Introduction

During the last decade, the treatment of cancer has been revolutionized by the widespread use of the immune checkpoint inhibitors (ICIs) monoclonal antibodies. Antibodies targeting Cytotoxic T lymphocyte-associated antigen-4 (CTLA-4), Programmed cell death 1 and Programmed cell death ligand 1 (PD1/PDL1) have been approved by the Food and Drug Administration and the European Medicine Agency for the treatment of a wide range of malignancies. The inhibition of these checkpoint proteins enhances the immune response against cancer cells, but at the same time decreases the immunological self-tolerance [1]. Thus, oncologists have to face several toxicities that differ from those induced by conventional cytotoxic chemotherapy or tyrosine kinase inhibitors [2]. Immune-related adverse events (irAEs) often affect the endocrine system [3] and immune-related thyroid diseases are frequent, particularly during anti-PD1 therapy [4]. Transient thyrotoxicosis and transient/permanent hypothyroidism are common thyroid toxicities [5–7], while Graves' disease is less frequent [8–10]. Many efforts have been made to identify potential risk factors for thyroid irAEs, but results are conflicting. Moreover, thyroid irAEs have been associated with a better overall survival, but it is unclear whether this is consequent to a longer exposure to ICI that in turn may expose the patient to an increased risk of autoimmune disorders [11, 12].

In this retrospective study, we analyzed the 195 subjects treated with ICIs for different metastatic tumors at a single center (1) to identify potential risk factors for thyroid irAEs and (2) to analyze the clinical outcome of subjects who developed thyroid dysfunctions compared to patients who remained euthyroid.

## Materials and methods

### Study population and objectives

**Fig. 1:**
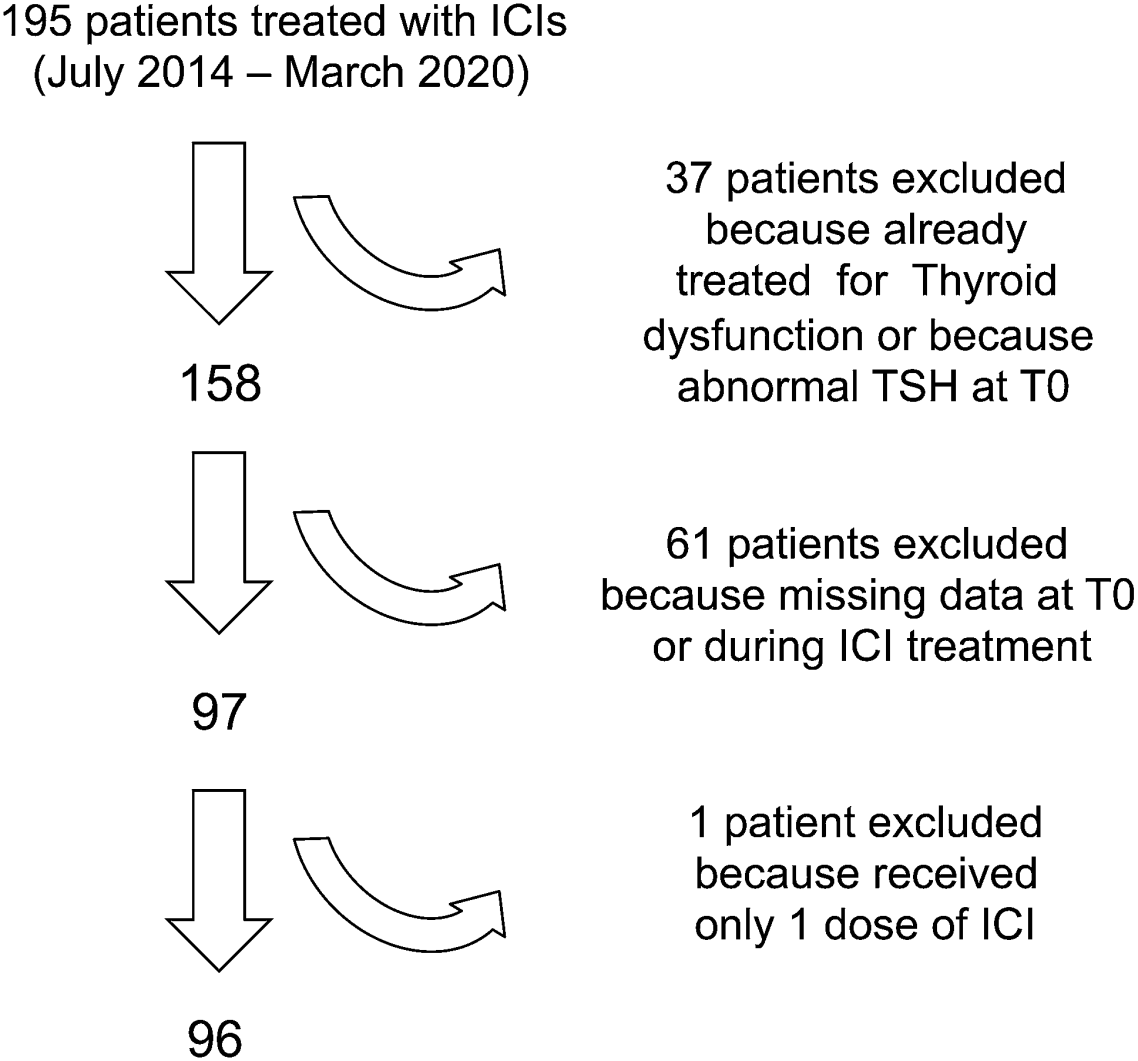
Patient selection and exclusion criteria

We retrospectively analyzed 195 consecutively patients who received ICIs for the treatment of a metastatic cancer at the Medical Oncology Department of the University Hospital "Federico II" of Naples between January 2014 and March 2020. Medical records were reviewed to obtain the following information: (1) demographic data (gender and age at the beginning of the therapy with ICIs), (2) cancer type and previous anti-neoplastic therapies including TKIs, (3) type and duration of ICI treatment (nivolumab, pembrolizumab, ipilimumab), (4) thyroid hormone status at baseline and during the ICI treatment, (5) date of cancer progression according to RECIST criteria v 1.1 and édate of death. The inclusion criteria were: (1) any kind of cancer treated with at least 3 courses of ICIs, (2) available serum thyroid hormone evaluation at baseline (3) availability of at least two different serum thyroid hormone evaluations during the ICI treatment, including serum determination of T3, T4 and TSH levels; the search for serum anti-thyroid antibodies (anti-thyroglobulin and anti-peroxidase) was recorded whenever performed; the dates of serum determinations were recorded. We excluded 18 subjects who had a history of thyroid disease and 19 who were not biologically euthyroid at baseline, 61 who had no thyroid hormone evaluation at baseline or during treatment, and 1 who received only one dose of ICI. A total of 96 subjects were included in our analysis (Fig. 1). During ICI treatment, patients were classified according to the TSH levels as (1) euthyroid (0.4 < TSH < 4 mIU/L), (2) hypothyroid (TSH > 4 mIU/L) and (3) hyperthyroid (TSH < 0.4 mIU/L). All thyroid hormone measurements were performed in the same laboratory.

### Statistical analysis

Statistical tests performed were: frequency analysis, the Chi Square test for the evaluation of the significance between the nominal variables, Mann–Whitney *U* test and Student’s *t* test for unpaired data to compare values and average of continuous variables, Kaplan Meier curves (Gehan-Breslow-Wilcoxon test) for the calculation of OS and PFS. A *p* value < 0.05 was assumed as statistical significance. The statistical analysis was performed using the Graphpad software 8.

## Results

### Patient characteristics

A total of 96 patients satisfied the inclusion criteria (Fig. 1), 69% were males and 72% were previously submitted to other systemic treatments. Their median age at the initiation of ICI treatment was 61 years (27–87); 43 (45%) had a non-small cell lung cancer (NSCLC), 31 (32%) a melanoma, 16 (17%) a renal cell carcinoma, and 6 (6%) another cancer type. Eighty-five patients (89%) were treated with an anti-PD 1 antibody (nivolumab 70% and pembrolizumab 19%). Nine patients (9%) who were affected by melanoma received ipilimumab. One patient was treated with ipilimumab combined with nivolumab and another patient with pembrolizumab in association with chemotherapy (pemetrexed plus cisplatin). All subjects with RCC were treated with nivolumab.

**Fig. 2:**
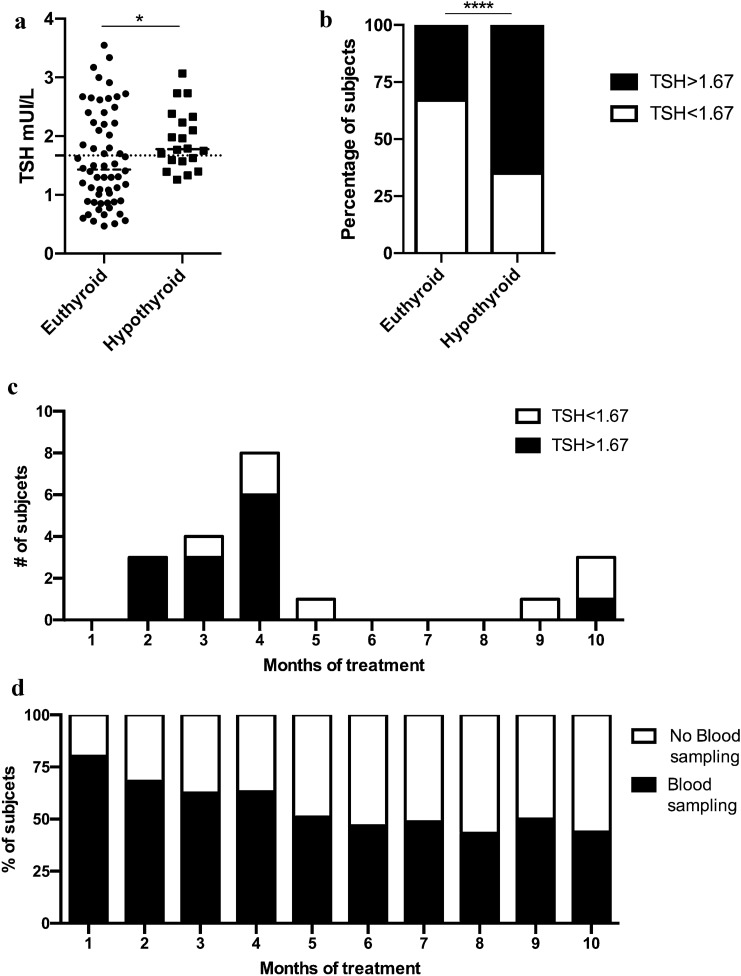
Baseline TSH levels are associated with thyroid irAEs. **a** Baseline TSH levels in subjects who remained euthyroid (circle) during the ICI treatment or became hypothyroid (square); the dot lines indicate the TSH cutoffs associated with an increased risk of hypothyroid irAEs **b** Percentages of subjects that had a TSH higher than 1.67 mIU/L (black) and lower 1.67 mIU/L (white) and who remained euthyroid or became and hypothyroid. **c** Timing of appearance of hypothyroid irAEs; white boxes indicate subjects with a baseline TSH lower than 1.67 mIU/L, black boxes indicate subjects with a baseline TSH higher than 1.67 mIU/L. **d** Percentage of blood sampling for thyroid hormones and TSH levels performed during the first 10 months of treatment; with open boxes, percentage of patients who have not been tested, black boxes percentage of subjects who have been tested

Thyroid hormone levels were tested at baseline and after treatment initiation at a median of once a month during the first 5 months of treatment and later at a median of every 2 months (Table 2 and Fig. 2d). All patients had at least one serum thyroid hormone measurement between month 6 and month 12. Baseline anti-thyroid antibody status (anti-thyroglobulin and anti-thyroperoxidase) was available only for 43 patients, 8 (19%) of whom were positive (Table 2).

### Baseline TSH level and positive anti-thyroid antibodies are predictive risk factors for the development of thyroid dysfunction

During ICIs treatment, 36 patients (38%) developed thyroid dysfunctions. The median time to first appearance of thyroid irAEs was 45 days (9–673 days). Twenty-five patients experienced a transient thyrotoxicosis, which progressed to hypothyroidism in nine patients and 11 experienced hypothyroidism without any recognized thyrotoxicosis. The median time of appearance of thyrotoxicosis was 38 days (9–673 days), 19 experienced the thyrotoxicosis within the first 2 months of treatment and six at a later time (Fig. 1S). The median time between the diagnosis of thyrotoxicosis and that of hypothyroidism was 57 days (27–249 days).

Twenty (21%) patients developed hypothyroidism that occurred later and was prolonged or even definitive in the majority of patients. The median time of appearance of hypothyroidism, since ICI treatment initiation was 106 days (32–189 days). Of these twenty patients, fifteen (75%) patients experienced the hypothyroid irAEs within the first 4 months of treatment, and only 5 (25%) at a longer time (one within 5 months, and the other 4 within 9 months of treatment; Fig. 2c).

No statistically significant differences were observed for gender, age at the initiation of ICI, number of doses or tumor type (NSCLC and melanoma) or ICI (Nivolumab and pembrolizumab) between subjects who remained euthyroid and those who developed hypothyroidism (Table 1). Interestingly, higher baseline TSH levels but that were still within the normal range, were observed in patients who developed hypothyroidism compared to those who remained euthyroid (*p* < 0.0025; Fig. 2a, Table 2). Accordingly, the ROC curve analysis demonstrated that TSH levels above 1.67 mIU/L were associated with an increased risk to develop hypothyroidism: (OR = 2.0, 95% CI 0.62–0.81, *p* = 0.0030) (Fig. 2b); 13/20 patients with a baseline TSH > 1.67 mIU/L became hypothyroid vs. 7/20 patients with a baseline TSH < 1.67 mIU/L. Moreover, 93% of hypothyroid cases in subjects with basal TSH higher than 1.67 mIU/L occurred within the first 4 months of treatment, and 33% of hypothyroid cases in the subjects with TSH lower than 1.67 mIU/L occurred during the same period of time (*p* = 0.0139; Fig. 2c).

**Table 1:** Patient characteristics at baseline and during ICI treatment

Characteristics	Baseline	Thyroid Status during ICI treatment			
	Overall	Euthyroid	Hypothyroid	OR	*p* value
Number of Subjects	96 (100%)	76	20		
Gender					
Female	30 (31.2%)	21 (27.6%)	9 (45%)	0.5	0.1651
Male	66 (68.8%)	55 (72.4%)	11 (55%)		
Age					
Diagnosis	59 (25–83)	59 (25–83)	62 (42–82)	0.9867	1.0000
Immunotherapy	61 (27–87)	61 (27–84)	63 (47–87)	1.052	0.8012
Primary tumor					
Squamous/NSCLC	43 (45.3%)	37 (48.7%)	6 (30%)	2.145	0.2376^b^
Melanoma	31 (32%)	23(30.3%)	8 (40%)		
RCC	16 (16.5%)	12 (15.8%)	4 (20%)		
Other	6 (6.2%)	4 (5.3%)	2 (10%)		
Immunotherapy agent					
Nivolumab	67 (69.1%)	49 (69.8%)	18 (90%)	0.3403	0.2187^c^
Pembrolizumab	18 (18.5%)	16 (18.8%)	2 (10%)		
Ipilimumab	9 (9.3%)	9 (9.4%)	0		
Other	2 (3.1%)	2 (2.1%)	0		

^a^AbT status was available only for 43 subjectecs

^b^Statistical analysis has been performed on Squamous/NSCLC and Melanoma groups

^c^Statistical analysis has been performed on Nivolumab and Pembrolizumab

**Table 2:** Thyroid status at baseline

Characteristics	Baseline	Thyroid status during ICI treatment			
	Overall	Euthyroid	Hypothyroid	OR	*p* value
Number of Subjects	96 (100%)	76	20		
Thyroid Hormone profile^a^	mUI/ml	pg/ml	ng/ml		
TSH	1.4 (0.5–3.5)	1.3 (0.5–3.5)	1.8 (1.3–3.1)	–	**0.0025**
FT3	3.0 (0.5–5.7)	2.9 (0.5–5.7)	3.0 (2.1–5.6)	–	0.8914
FT4	1.2 (0.7–2.2)	1.2 (0.7–2.1)	1.2 (0.8–2.2)	–	0.7557
AbT^b^					
Negative	35 (81.4%)	32 (94.1%)	6 (66.7%)	**32.0**	**0.0003**
Positive	8 (18.6%)	2 (5.9%)	3 (33.3%)		

^a^Median, minimum and maximum values have been reported

^b^Number of subjects and percentage of subjects have been reported

Six of the eight subjects with positive anti-thyroid antibodies (AbT) at baseline developed hypothyroidism, whereas only 3 of the 35 subjects with negative baseline AbT became hypothyroid (*p* < 0.0001; Table 2). Five of six patients with positive AbT who became hypothyroid had a baseline TSH higher than 1.67 mIU/mL (Table 2).

### Progression-free survival and overall survival

**Fig. 3:**
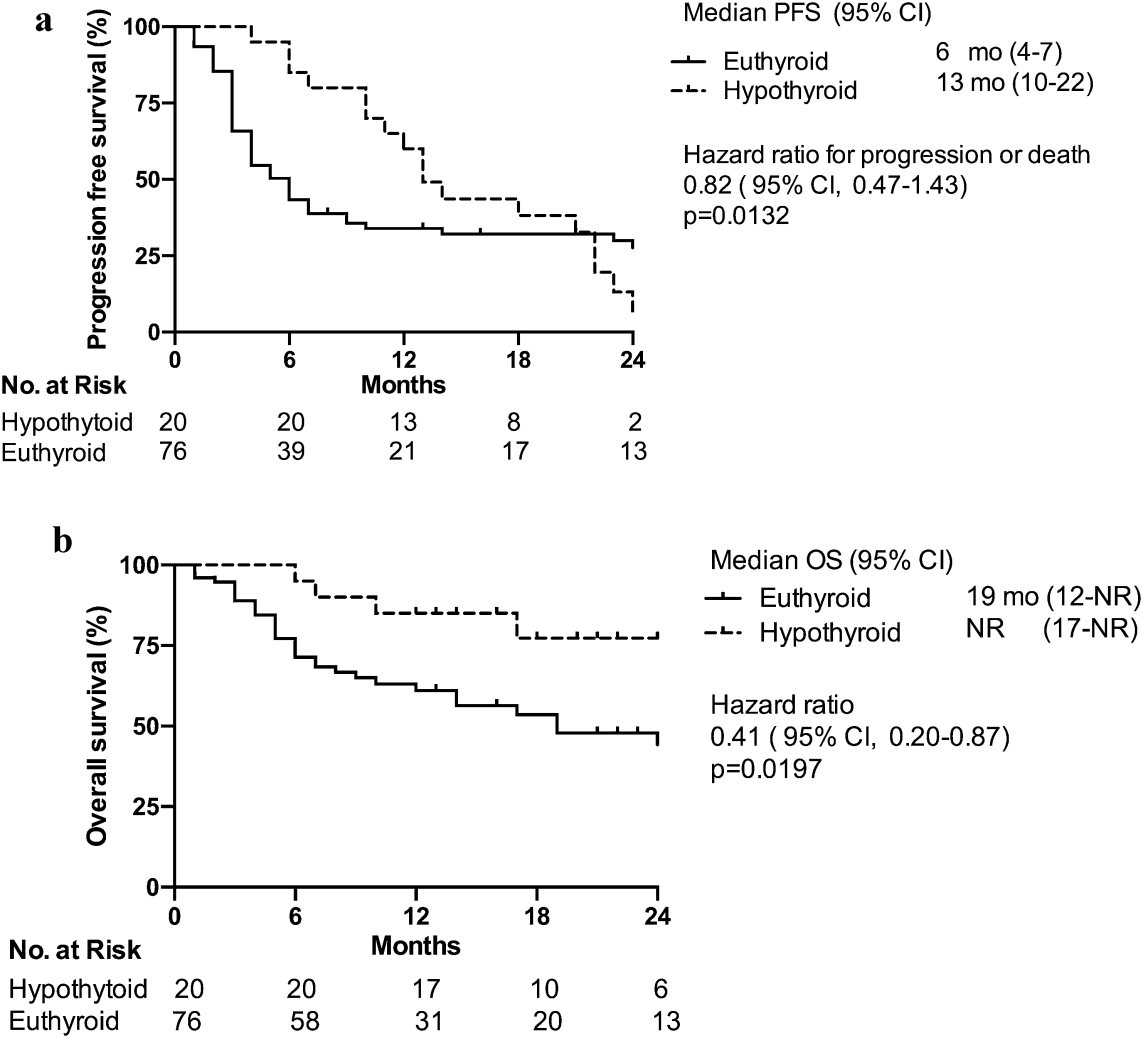
Thyroid irAEs correlate with ICI efficacy. **a** Kaplan–Meier curve of PFS of euthyroid (black line) and hypothyroid irAEs subjects (dotted line). **b** Kaplan–Meier curve of OS of euthyroid (black line) and hypothyroid irAEs subjects (dotted line)

After a median follow-up after initiation of ICI treatment of 11 months (1–69), the median PFS was 7 months and the median OS was 25 months. In subjects with hypothyroidism irAEs, the median PFS 13 months compared with 6 months for patients who remained euthyroid (HR = 0.82 CI 0.47–1.43; *p* = 0.0132; Fig. 3a). In the hypothyroid irAE group, the median OS was not reached compared with 19 months for the euthyroid patients (HR = 0.41 CI 95% 0.20–0.87; *p* = 0.0197; Fig. 3b).

Although baseline TSH levels above 1.67 mIU/L were risk factors for thyroid irAEs, they were not related to a better survival as compared to lower levels (NR vs. 24 months, *p* = 0.760; (Fig. 1S).

## Discussion

In this study, we demonstrated that higher TSH levels and anti-thyroid antibody status at baseline represent predictive biomarkers for hypothyroidism during ICI treatment. Furthermore, patients who developed thyroid irAEs had an improved PFS and an improved OS.

Baseline TSH levels were able to predict the appearance of the irAEs. Hypothyroidism occurred more frequently and earlier in patients with a baseline TSH level above 1.67 mIU/L.

In most cases, thyroid irAEs do not require ICI interruption or administration of high doses of corticosteroids; however, it is important to perform early diagnosis, because overt hypothyroidism or hyperthyroidism may severely impair patients’ clinical conditions and quality of life [2, 13].

In our cohort, the incidence of thyroid irAEs was similar to that observed in previous studies [2]. Hypothyroidism was the most frequent thyroid irAE and in nine subjects was preceded by a transient hyperthyroidism, consistent with the biochemical course of a thyroiditis. In the other 11 patients who apparently developed directly hypothyroidism, we might have missed the transient hyperthyroid phase, due to the retrospective nature of our study with long intervals of time between blood measurements in some patients. Differently to what is observed in autoimmune thyroid diseases, women and men are equally affected by thyroid irAE [14, 15]. Previous studies showed that in patients treated with ICIs higher baseline TSH levels (namely, > 2.19 mIU/L and 1.72 mUI/ml) are associated with increased risk for thyroid irAEs [16, 17]. This is consistent with our findings, although our TSH cut off value is lower (i.e., 1.67 mIU/L). In our study, thyroid function evaluation has been performed every 4–6 weeks for the first 3 months in accordance with the ASCO guidelines. Although, thyroid hormone measurements were not performed at each cycle of treatment, the rate of hypothyroidism in this study is similar to those observed in other previous studies. Therefore, for this reason, we do not believe that the missing TSH measurement could strongly affect our results.

Previous studies showed that AbT at baseline are associated with an increased risk of thyroid irAEs [18]. In our study, only eight patients had a documented positive AbT status, and 6 of them developed hypothyroidism. A recent study by Brilli et al. showed that the shift of AbT from negative to positive during the ICI treatment was associated with a higher rate of overt thyroid dysfunction but, interestingly, the switch occurred after the onset of the thyroid dysfunction [17]. However, autoimmune thyroiditis might be present without detectable AbT and might be responsible for higher serum TSH levels that in turn might be the basis of the increased incidence of hypothyroidism in such patients. In future, prospective studies will be needed to clarify the putative cause-effect correlation between AbT and hypothyroidism development during ICI treatment, and also to investigate other important aspects as thyroid ultrasonography features.

The better median PFS and OS in patients with thyroid irAEs is in line with previous studies and [19, 20], and it could be related to a different immunological background. Subjects that developed hypothyroidism could have an immunological background that exposes them to a higher risk of thyroid irAEs but at the same time that makes them more sensitive to the ICI treatment. The presence of AbT at baseline might be a marker of this background.

In conclusion, on the basis of our results, in accordance with the ESMO and ASCO guidelines, we suggest to test thyroid hormone and TSH levels at baseline and every 4–6 weeks for the first 3–6 months of treatment in all patients and with a particular caution in patients with baseline TSH > 1.67 mIU/L and/or with positive AbT.

## Supplementary Information

Below is the link to the electronic supplementary material.

Supplementary file1 (TIFF 170123 KB)

Supplementary file2 (TIFF 170123 KB)

Supplementary file3 (DOCX 36 KB)
