# Supplementary material for: Higher baseline TSH levels predict early hypothyroidism during cancer immunotherapy
Source: J Endocrinol Invest. 2021 Feb 12;44(9):1927–33. doi: 10.1007/s40618-021-01508-5 (PMC8357750; doi:10.1007/s40618-021-01508-5)
Supplement: Supplementary file 3 — Supplementary file3 (DOCX 36 KB) [file 40618_2021_1508_MOESM3_ESM.docx]

**Supplemental figure legends**

**Fig1S** Timing of appearance of thyrotoxicosis irAEs

**Fig2S**  Kaplan-Meier curve of OS relative to subjects with baseline TSH levels either lower than 1.67 mIU/L (black line) or higher than 1.67 mIU/L (dotted line).
